# Maternal Diets Trigger Sex-Specific Divergent Trajectories of Gene Expression and Epigenetic Systems in Mouse Placenta

**DOI:** 10.1371/journal.pone.0047986

**Published:** 2012-11-05

**Authors:** Anne Gabory, Laure Ferry, Isabelle Fajardy, Luc Jouneau, Jean-David Gothié, Alexandre Vigé, Cécile Fleur, Sylvain Mayeur, Catherine Gallou-Kabani, Marie-Sylvie Gross, Linda Attig, Anne Vambergue, Jean Lesage, Brigitte Reusens, Didier Vieau, Claude Remacle, Jean-Philippe Jais, Claudine Junien

**Affiliations:** 1 INRA, UMR1198 Biologie du Développement et Reproduction, Jouy-en-Josas, France; 2 L’Ecole Nationale Vétérinaire d’Alfort (ENVA), Maisons Alfort, France; 3 Inserm; AP-HP; Université Paris-Descartes, Faculté de Médecine, Hôpital Necker-Enfants Malades, U781, SBIM, Paris, France; 4 EA 4489 Unité Environnement Périnatal et Croissance, Department of Diabetology, Biology and Pathology Center, Huriez Hospital, CHRU Lille, Lille, France; 5 EA 4489 Unité Environnement Périnatal et Croissance, Université de Lille 1, Bâtiment SN4, Villeneuve d’Ascq, France; 6 Laboratory of Cell Biology, Institute of Life Sciences, Université catholique de Louvain, Louvain-la-Neuve, Belgium; 7 Service de Biostatistique et Informatique Médicale, Hôpital Necker-Enfants Malades, Paris, France; 8 Université de Versailles Saint-Quentin-en-Yvelines, Versailles, France; State University of Rio de Janeiro, Brazil

## Abstract

Males and females responses to gestational overnutrition set the stage for subsequent sex-specific differences in adult onset non communicable diseases. Placenta, as a widely recognized programming agent, contibutes to the underlying processes. According to our previous findings, a high-fat diet during gestation triggers sex-specific epigenetic alterations within CpG and throughout the genome, together with the deregulation of clusters of imprinted genes. We further investigated the impact of diet and sex on placental histology, transcriptomic and epigenetic signatures in mice. Both basal gene expression and response to maternal high-fat diet were sexually dimorphic in whole placentas. Numerous genes showed sexually dimorphic expression, but only 11 genes regardless of the diet. In line with the key role of genes belonging to the sex chromosomes, 3 of these genes were Y-specific and 3 were X-specific. Amongst all the genes that were differentially expressed under a high-fat diet, only 16 genes were consistently affected in both males and females. The differences were not only quantitative but remarkably qualitative. The biological functions and networks of genes dysregulated differed markedly between the sexes. Seven genes of the epigenetic machinery were dysregulated, due to effects of diet, sex or both, including the Y- and X-linked histone demethylase paralogues *Kdm5c* and *Kdm5d,* which could mark differently male and female epigenomes. The DNA methyltransferase cofactor *Dnmt3l* gene expression was affected, reminiscent of our previous observation of changes in global DNA methylation. Overall, this striking sexual dimorphism of programming trajectories impose a considerable revision of the current dietary interventions protocols.

## Introduction

According to the ‘Developmental Origins of adult Health and Disease’ (DOHaD) concept, adverse environment during the developmental program may affect the long-term health and susceptibility to non communicable diseases of the offspring [1]. The effects of maternal nutrient deprivation have been well-characterized. However, there are less studies examining the potentially deleterious effects of maternal overnutrition or metabolic disturbances on the future health of the offspring, particularly as concerns the development of a typical metabolic syndrome phenotype i. e. obesity, type 2 diabetes (T2D), cardiovascular diseases (CVD) [2], [3], [4], [5]. In mice, maternal obesity, T2D and a high-fat diet (HFD) during gestation increase adiposity and modify metabolism and blood pressure in adult offspring fed a control diet (CD), revealing a predisposition to the development of metabolic syndrome [6], [7]. Given the large and increasing proportion of women of child-bearing age that are overweight and eat inappropriately during pregnancy, there is an urgent need to offer advice and evidence-based dietary recommendations, clinical treatment and counseling to these women and their babies.

The placenta plays a key role in the mother-fetus relationship, maintaining fetal homeostasis through the regulation of nutrient transfer. Changes in placental structure, activity or physiology therefore trigger an adaptive response, supporting the involvement of placenta in programming, e.g. of CVD [8], [9], [10]. Both the development and function of the placenta are subject to dynamic regulation by environmental factors, including nutrient status and tissue oxygenation [11], [12], [13]. The placenta is thus an appropriate organ to study the ways in which unbalanced maternal food consumption is sensed by the developing fetus [14], [15].

The effects of maternal overnutrition on the long term consequences on metabolic disturbances in the offspring is now increasingly documented in the literature on DOHaD. However, despite the major role of the placenta in materno-fetal exchanges and the ease with which large amounts of placental tissue may be sampled, the effects of maternal nutritional status on the development of the placenta and its role in nutritional adaptive processes is still poorly understood, particularly concerning overnutrition. The data obtained in rodent and large animal models suggest that placental development is highly adaptable and that many types of compensation for suboptimal nutrition are possible [16], [17], [18], [19], [20].

The placenta has long been considered as an asexual organ, with most studies consistently pooling data derived from the placentas of male and female fetuses into a single group [21]. However, as predisposition to adult diseases, such as T2D, hypertension and CVD is shaped by what the mother consumes during her pregnancy, differences in the frequency of these diseases between the sexes are probably mediated also by sex-specific placental functions. There is growing evidence that the sex of the placenta plays a major role in determining fetal size, nutrition, morbidity and survival [17], [21]. Molecular investigations of the similarity or difference between the responses of male and female conceptuses to the same maternal diet are therefore of interest. Several groups have reported differences in gene expression and protein levels for several pathways that may potentially influence the outcome of several challenges during pregnancy (e.g. genes encoding Insulin-like growth factors and Cytochrome P450 (*CYP1A)*, proteins involved in glucocorticoid synthesis, Epidermal growth factor pathways) [22], [23], [24]. However, few groups have studied global sexual dimorphism in the placenta through the use of microarrays, addressing, in particular, the impact of maternal diet on placental gene expression, through systematic investigations of the relationship between diet and the expression of sexually dimorphic genes [17], [25].

There is convincing experimental evidence to suggest that epigenetic marks serve as a memory of exposure to inappropriate environments, thus potentially affecting fetal and placental programming [26]. Developmental alterations to these marks can induce long-term changes in gene expression, potentially leading to disease in later life [27], [28]. In a previous report, we compared the gene expression patterns of 20 imprinted genes and analyzed global DNA methylation, in placentas from male and female mouse fetuses with mothers fed a HFD or a CD, in the middle of the fetal period, when the morphological development of the placenta is complete and fetal growth is maximal [29]. A HFD during gestation triggers the sex-specific deregulation of clusters of imprinted genes, including the *Igf2r* cluster, which plays an important role in the control of many cellular, metabolic and physiological functions potentially involved in adaptation and/or evolution. Sex-specific differences in global DNA methylation and changes to the methylation of specific CpGs in the *Igf2r* DMR were observed.

Using the same model, in the present study, we investigated the effects of maternal diet on gene expression and epigenetic marks at the whole-genome level. We used the Affymetrix mouse exon microarray to investigate the impact of a HFD during pregnancy in male and female mouse placentas. This analysis revealed differential expression patterns in CD and HFD groups for genes corresponding to different functions, this pattern being dominated by sexual dimorphism. Moreover, the expression of genes belonging to the epigenetic machinery was sexually dimorphic and altered by HFD.

## Materials and Methods

### Experimental Design and Nutritional Treatments

All experiments on animals were conducted in accordance with the European Communities Council Directive of 1986 (86/609/EEC). Our laboratory holds accreditation from the French Ministry of Agriculture for experimentation with mice (A75-15-02). The animal model was previously described [29]. The protocol referred to above received a favorable opinion (visa 11/044) from the COMETHEA ethical committee (Comité d’éthique pour l’expérimentation animale), registered with the national Comité National de Réflexion Ethique sur l’Expérimentation Animale under the n°45.

### Histological Analysis

Classical hematoxylin-eosin (HE) staining was performed on the largest section of paraffin embedded placenta for four placentas per group. As the largest and smallest placentas in a litter have been shown to be morphologically different in mice [30], we used the female and male placentas corresponding to the median weight. Placentas were fixed by incubation overnight in 4% PFA in phosphate-buffer 0.1 M pH 7.6, dehydrated in ethanol, embedded in paraffin wax and cut into 7 µm sections. The largest slide of each placenta was stained with hematoxylin and eosin (HE) and imaged with Nanozoomer (Hamamatsu) at x40 resolution. The area and the major/minor lengths of the labyrinth and total placenta were calculated with ImageJ software.

### RNA Extraction

Total RNA was extracted from rodent placenta with the RNeasy Mini kit, according to the manufacturer’s instructions (Qiagen). Concentrations were estimated by measuring absorbance at 260 nm with a spectophotometer (Nanodrop).

### Microarray Data Analysis

We pooled 5 µg of RNA per sample according to sex and diet, for each litter, so as to obtain four groups: F CD (n = 4), M CD (n = 4), F HFD (n = 4) and M HFD (n = 4). RNA concentrations were normalized to 100 ng/µl. Microarray hybridization was performed by PartnerChip (Evry, France). RNA integrity number and 28S/18S ratio were determined by the Agilent chip (2100 Bioanalyzer). mRNA enrichment was achieved with oligo-d(T) magnetic beads. cDNAs were synthesized with SuperScriptII (Invitrogen), purified and fragmented. At each step Agilent chip quality control was carried out. The 16 cDNA samples were labeled with biotin and hybridized onto 16 Affymetrix Mouse Exon 1.0 ST microarrays. Binding was detected by incubation with phycoerythrin-conjugated streptavidin (GeneChip 450 fluidic station). Fluorescence was read with a GeneChip 3000 scanner equipped with GCOS software (v1.4). Quality control was performed with Expression Console software (Affymetrix).

#### Affymetrix assay and data processing

We used the Affymetrix Mouse Exon 1.0 ST microarray, which carries 1.2 million probe sets covering one million exon clusters, with an average of 40 probes per gene. Raw data were processed for background correction and normalization with the apt-probeset-summarize program from the Affymetrix Power Tools (APT v1.6) and the array definition files available on the Affymetrix web site. RMA algorithm with full quantile normalization was applied using the array design library files V1 release 2 and core-gene definition files based on mouse genome release mm8 defining 23,238 probesets. Transcripts were annotated with the Affymetrix MoEx-1.0-st transcript cluster annotation file release 23. Transcripts annotated as control or normalisation genes were discarded letting 16,654 genes for further analyses. Differential expression profiling analyses were performed with the R Bioconductor LIMMA package [31], [32]. Raw *p*-values and adjusted *p*-values (FDR) were calculated. Considering the limited number of differentially expressed genes with FDR, we decided to work with the raw *p*-values and to validate with RT-qPCR the most important candidate genes. The data have been deposited in NCBI’s Gene Expression Omnibus [33] and are accessible through GEO Series accession number GSE29585 (http://www.ncbi.nlm.nih.gov/geo/query/acc.cgi?acc=GSE29585).

#### Ingenuity pathway analysis

The differentially expressed gene datasets were uploaded into IPA software (content version 11904312) and a core analysis was performed for each comparison: F HFD *vs* F CD, M HFD *vs* M CD, genes common to analyses of female and male placentas, M CD *vs* F CD, M HFD *vs* F HFD, genes common to CD and HFD analysis. The Ingenuity Core Analysis gives 3 types of information: biological functions associated to the genes in dataset, network of gene in dataset and canonical pathways associated to the genes in dataset. For each core analysis, we chose to limit our analysis to the first 10 functions (with the exclusion of disease functions), and to networks with a score ≥25. Finally, due to the small number of genes for each pathway, the canonical pathway categories had very small ratios and this result was not taken into account.

### Reverse-transcription and Quantitative PCR (RT-qPCR)

The RT-qPCR experiments were performed as previously described [29], with exception of the normalisation method. *18S, Eif4a2, Actß, Tbp, Gapdh, Mrpl32, Tfrc* and *Sdha* were tested as reference genes in GeNorm software [34]. We normalized the qPCR results with the normalization factor obtained for the *Eif4a2*, *Tbp* and *Sdha* quantifications. The -RT controls tested for the eight reference genes assessed the absence of gDNA contamination for each sample. Primer sequences and qPCR assay conditions are available upon request.

### Histone Preparation and Western Blotting

Histones were prepared from 25 mg of total placenta from 10 individual samples for each group, as described [35]. Protein concentration was determined with the Pierce BCA assay kit (ThermoScientific). We pooled 2 µg of protein per sample together as a function of sex and diet, to obtain four groups: F CD, M CD, F HFD and M HFD. Histones (2 µg) were separated by SDS-PAGE in a 15% acrylamide gel and transferred to a PVDF membrane (0.2 µm; Millipore). The membrane was blocked by incubation with 5% nonfat milk in 0.1% Tween 20 in PBS (PBS-Tween). The membrane was incubated overnight with primary antibody in 2% nonfat milk (anti-H3K9me3 1:500, anti-H3K4me3 1:1000, Millipore 07–442 and 07–473), washed in PBS-Tween and then for 1hour with horseradish peroxidase-conjugated secondary antibody (anti-rabbit IgG 1∶10000, Sigma A6667). The signal was detected with ECL Plus (Amersham) in LAS-1000 (Fujifilm). The membrane was stripped and incubated for 1 hour with anti-pan H3 antibody (Abcam ab1791, 1∶5000). The amount of trimethylated H3 was quantified with normalization to the amount of total H3 in each pool (AIDA Software).

### Statistical Analysis

All data are expressed as means ± standard error of the mean (SEM). The effects of sex and diet on histological measurements for the placenta, RT-qPCR and western-blot data were assessed by non-parametric Kruskal-Wallis test, with Mann and Whitney post-hoc test. Correlation between microarray and RT-qPCR data was assessed with Spearmann correlation test. All tests were performed with StatEl software (AD Science).

## Results

### Histological Analysis

We previously showed on the same cohort that there was a main effect of maternal HFD and sexual dimorphism on placental weight and fetal weight to placenta weight index (FPI) [29]. In order to assess whether these weight changes were associated with morphological changes we analysed the placenta with classical histological approaches. No obvious changes were observed in the structure of the labyrinth or spongiotrophoblast on haematoxylin-eosin-stained slides: the number and size of the cells and vessels were identical in the four groups (Supporting Figure S1-A and B). The proportion of the placenta occupied by the labyrinth was not affected by diet or sex of the offspring, Moreover, overall placental and labyrinth shapes, estimated from minor and major lengths, did not differ between the four groups (Supporting Figure S1-C). Thus, neither the sex of the offspring nor HFD in the mother triggered gross morphological changes in the structure and size of the placental layers.

### Transcriptomic Analysis

**Figure 1:**
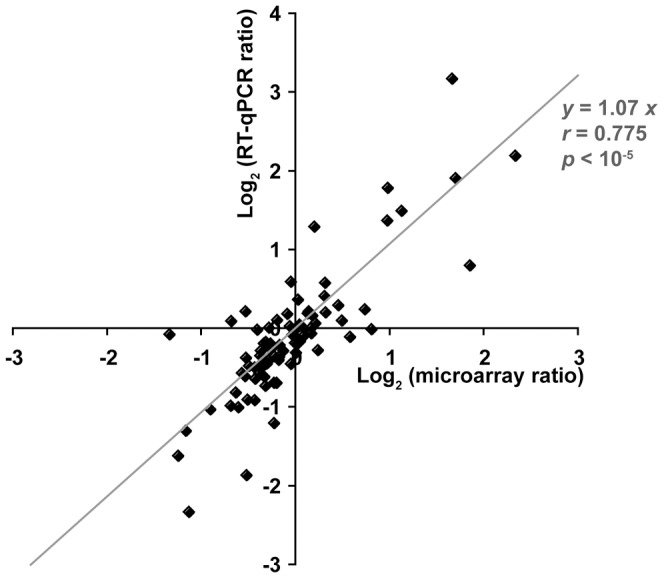
A plot of the RT-qPCR and microarray values. for the gene expression coefficient (log2 of ratios) for the 4 different comparisons (F HFD vs F CD, M HFD vs M CD, M CD vs F CD and M HFD vs F HFD) for 21 genes (*Hsd11b1, Hsd11b2, Cxcl1, Prap1, Slc13a4, Slc22a3, Sult1e1, Tph1, Gcm1, Jarid1c, Suv39h1, Suv39h2, Dnmt3l, Maoa, C3, Pdgfß, Drd4, Vdr, Prl7a1, Gzmb, CD81*). The RT-qPCR data correlated with the microarray data for most of the 84 coefficient, with a linear equation: *y = *1.07x and a high correlation coefficient (*r = *0.775). Spearman’s test indicated that this correlation was highly significant (*p*<10-5).

We used LIMMA statistical analysis (Linear Models for MicroArray data) to evaluate the effect of diet and sex on gene expression in our placenta samples (Table 1 and Limma tables are available in supporting table S1). The microarray data were validated by performing RT-qPCR analysis on six litters from CD mothers and seven litters from HFD mothers, for 21 genes. These genes were selected either for their specific functional interest or because they are actors of the functions or network subsequently described or because they were dysregulated in both sexes. Expression coefficients (log_2_ of expression ratio) for each comparison, obtained by RT-qPCR (*y*-axis), were plotted against equivalent values from the microarray data (*x*-axis). In general, the RT-qPCR and microarray data were consistent, for most of the 84 coefficients, as indicated by the statistical significance of the linear regression equation: *y* = 1.07*x* (Spearman correlation, *p*<10^−5^, *r* = 0.775), highlighting the robustness of the microarray data (Figure 1).

**Table 1:** Summary of disregulated gene numbers in Limma statistical analysis and Ingenuity pathway analysis.

Statistical comparison	Nb of disregulated genes		Nb of genes mapped by Ingenuity		
Sexual dimorphism	(*p*<0.05)	(*p*<0.01)	total	F>M	M>F
M CD *vs* F CD	**576**	**104**	97	54	43
M HFD *vs* F HFD	**646**	**97**	93	46	47
common			11	4	6

Diet effect	(*p*<0.05)	(*p*<0.01)	total	up regulated	down regulated
F HFD *vs* F CD	**888**	**168**	164	99	65
M HFD *vs* M CD	**875**	**190**	187	86	101
common			16	3	12

We obtained expression data for four groups: females (F) or males (M) placentas from mother under control (CD) or high-fat (HFD) diet (F CD, F HFD, M CD and M HFD). First, we analysed the data to detect sexual dimorphism in gene expression. For this purpose, we compared the data between males and females, under each nutritional condition. We therefore had two comparisons: M CD vs F CD and M HFD vs F HFD. Second, in order to describe the placental response to a maternal HFD challenge, we subsequently compared the HFD to the CD data in each sex: the two comparisons were F HFD vs F CD and M HFD vs M CD. Altogether, for these four comparisons, a bioinformatic analysis of functions and networks was carried out using the Ingenuity Pathway Analysis (IPA) software. IPA assigns functions to the genes and the Ingenuity Knowledge Base assesses the closeness of connections at the molecular level thereby defining networks. Considering the important number of gene differentially expressed for *p*≤0.05, which can include false positives and disturb the analysis, the datasets for IPA take into account genes for which *p*≤0.01.

#### Placental gene expression is sexually dimorphic, under CD and under HFD

**Figure 2:**
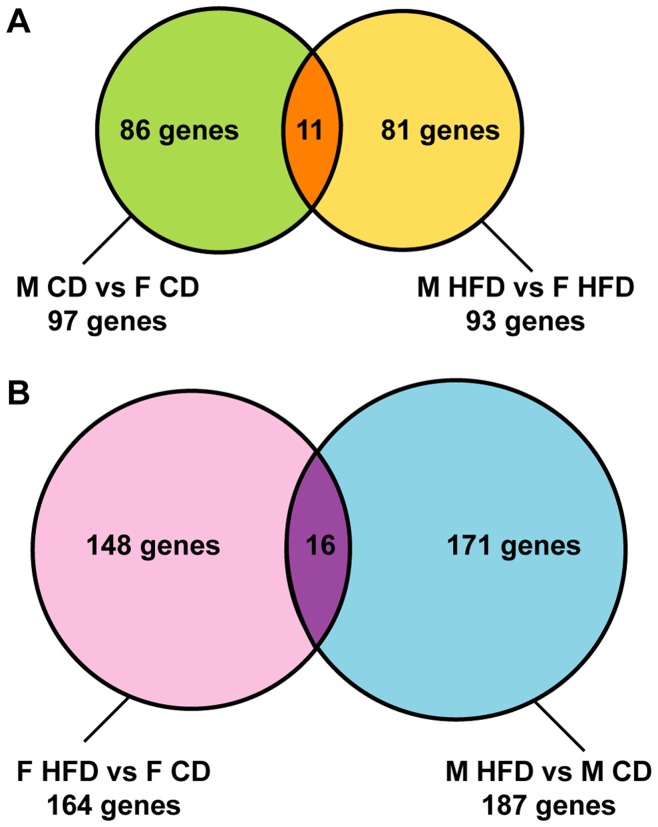
Venn diagram representing the number of genes in LIMMA statistical analysis. (A) displaying significant sexual dimorphism or (B) significantly dysregulated under the influence of maternal high-fat diet.

We first investigated whether there was a basal sexual dimorphism between female and male placentas from mothers fed a CD during pregnancy. We detected 104 genes displaying sexual dimorphism, of which 97 were mapped in IPA: 54 with higher expression in female than in male placentas and 43 with higher expression in male than in female placentas (Table 1 and Figure 2A). In the IPA results, the most relevant functions associated with sexually dimorphic genes under basal condition concerned chemotaxis of leukocytes, myeloid cells and phagocytes and adhesion of immune cells. Other functions were nervous system development and function, free radical scavenging, amino acid and carbohydrate metabolisms (Table 2). Network analysis revealed that interacting genes were associated with cell signaling, small molecule biochemistry, cell death for the first; nucleic acid metabolism, small molecular biochemistry, antimicrobial response for the second and cellular movement, cancer, tumor morphology for the last (Table 3).

**Table 2:** Ingenuity pathway analysis for sexually dimorphic genes: Biological functions under CD conditions.

Category and Functions Annotation a	*p-value*	nb gene	regulationz-score	Molecules
**Cellular Development**	***9,17E-04-4,87E-02***	**13**		
spermatogenesis	*1,77E-02*	4	2,135	Ddx3y ^m^, EAF2^m^, Eif2s3y ^m^, KDM5D ^m^
**Nervous System Development and Function**	***9,17E-04-4,87E-02***	**18**		
**Free Radical Scavenging**	***1,5E-03-1,24E-02***	**4**		
**Cell-To-Cell Signaling and Interaction**	***2,63E-03-4,71E-02***	**15**		
adhesion of immune cells	*4,71E-02*	4	1,469	*ANGPT1* f, FPR1 ^m^, *GPR132* f, *PIK3CG* f
**Cellular Movement**	***3,57E-03-4,48E-02***	**7**		
chemotaxis of mononuclear leukocytes	*3,57E-03*	4	0,009	*ANGPT1* f, FPR1 ^m^, *PIK3CG* f, *S100B* f
chemotaxis of cells	*1,12E-02*	6	−0,784	ANGPT1^f^, FPR1 ^m^, *GPR132* f, *HBEGF* f,*PIK3CG* f, *S100B* f
chemotaxis of myeloid cells	*1,24E-02*	4	0,121	*ANGPT1* f, FPR1 ^m^, *PIK3CG* f, *S100B* f
chemotaxis of phagocytes	*1,45E-02*	4	0,119	*ANGPT1* f, FPR1 ^m^, *PIK3CG* f, *S100B* f
**Hematological System Development and** **Function**	***3,57E-03-4,87E-02***	**11**		
chemotaxis of mononuclear leukocytes	*3,57E-03*	4	0,009	*ANGPT1* f, FPR1 ^m^, *PIK3CG* f, *S100B* f
chemotaxis of myeloid cells	*1,24E-02*	4	0,121	*ANGPT1* f, FPR1 ^m^, *PIK3CG* f, *S100B* f
chemotaxis of phagocytes	*1,45E-02*	4	0,119	*ANGPT1* f, FPR1 ^m^, *PIK3CG* f, *S100B* f
hematopoiesis	*4,56E-02*	7	−2,455	*ANGPT1* f, EAF2^m^, KDM5D ^m^, *NKAP* f, *OGT* f,*PIK3CG* f, *TNFRSF11A* f
adhesion of immune cells	*4,71E-02*	4	1,469	*ANGPT1* f, FPR1 ^m^, *GPR132* f, *PIK3CG* f
**Immune Cell Trafficking**	***3,57E-03-4,71E-02***	**5**		
chemotaxis of mononuclear leukocytes	*3,57E-03*	4	0,009	*ANGPT1* f, FPR1 ^m^, *PIK3CG* f, *S100B* f
chemotaxis of myeloid cells	*1,24E-02*	4	0,121	*ANGPT1* f, FPR1 ^m^, *PIK3CG* f, *S100B* f
chemotaxis of phagocytes	*1,45E-02*	4	0,119	*ANGPT1* f, FPR1 ^m^, *PIK3CG* f, *S100B* f
adhesion of immune cells	*4,71E-02*	4	1,469	*ANGPT1* f, FPR1 ^m^, *GPR132* f, *PIK3CG* f
**Antigen Presentation**	***3,92E-03-3,28E-02***	**6**		
chemotaxis of phagocytes	*1,45E-02*	4	0,119	*ANGPT1* f, FPR1 ^m^, *PIK3CG* f, *S100B* f
**Amino Acid Metabolism**	***4,15E-03-4,08E-02***	**2**		
**Carbohydrate Metabolism**	***4,15E-03-4,48E-02***	**7**		

a The first 10 biological categories of function are indicated and function annotation with a significant z-score are detailed.

f Genes more expressed in females. ^m^Genes more expressed in males.

Underline corresponds to genes both dimorphic under CD or HFD conditions.

**Table 3:** Ingenuity pathway analysis for sexually dimorphic genes: Networks under CD conditions (M CD *vs* F CD).

ID	Score^a^	Top Functions/Molecules in Network^b^
1	44	**Cell Signaling, Small Molecule Biochemistry, Cell Death**
		**ADRA1A** f **,** Akt, **ANGPT1** f **,** CD3, **CKS1B** ^m^ **,** **DDT** ^m^ **,** **EHD4** f **,** ERK, ERK1/2, **GAP43** f **,** Gpcr, **GSTM1** ^m^ **,** **HBEGF** f **, ID4** f **,** Jnk, **KDM5D** ^m^ **,** **KPNA2** f **,** Mapk, **MTHFD2** f **,** NFkB (complex), **OAZ1** f, **OGT** f **,** P38 MAPK, p70 S6k, PI3K (complex), **PIK3CG** f **,** Pkc(s), **PPP2R2C** ^m^ **,** Ras, RNA polymerase II, **S100B** f **, SLC2A12** f **, TAP2** ^m^ **,** **TNFRSF11A** f **,** Vegf
2	26	**Nucleic Acid Metabolism, Small Molecule Biochemistry, Antimicrobial Response**
		ABR, **B630019K06Rik** f **,** CLEC4E, CMPK2, DDX41, **Ddx3y** ^m^, DLG4, **EAF2** ^m^ **,** **Eif2s3y** ^m^, **ENPP3** ^m^ **,** GC, **Gm10696** ^m^, GUCY, **GUCY1A2** ^m^ **,** GUCY1B2, GUCY1B3, GUCY2E, GUCY2F, IFI44, **IFIT1B** f **,** IFNB1, IKBKG, **IL28RA** f **,** IRG, LMNA, **NKAP** f **,** **NPM3** ^m^ **,** OASL, **OLFML2B** f **,** RNPS1, SMURF1, **TCEANC** f **,** TNF, TNFAIP2, **ZBTB8B** f
3	25	**Cellular Movement, Cancer, Tumor Morphology**
		APLNR, ARRDC3, beta-estradiol, **BHLHA15** ^m^ **,** **C6orf125** ^m^ **,** CCKAR, CXCR7, EGFR, EGFR ligand, **EIF2S3** f **,** **FPR1** ^m^ **,** FPR, FYN, G protein, Gpcr, **GPM6B** f **,** GPR44, **GPR119** f **,** **GPR132** f **,** Gαq, IKBKE, LMNA, MYC, NFKBIA, **PGAP1** f **,** PIK3R5, PIK3R6, PLC, RGS1, **RNF125** f **,** **SPRR3** ^m^ **,** **SUV39H2** ^m^ **,** **TAL2** f **,** UBC, **UQCR10** ^m^

a Networks with scores ≥25 are indicated. ^b^Genes in bold are included in our datasets.

f Genes more expressed in females. ^m^Genes more expressed in males.

Underline corresponds to genes both dimorphic under CD or HFD conditions.

We then investigated whether sexual dimorphism was also observed when the mother was fed a HFD during pregnancy, by comparing the M HFD and F HFD placenta groups. We found that 97 genes were differentially expressed in the HFD placentas, of which 93 were mapped in IPA: 46 with higher expression in female than in male placentas and 47 with higher expression in male than in female placentas (Table 1 and Figure 2A). The functions associated with these genes concerned cell cycle, organ development, cellular compromise, growth of cell type (neurites or tumor) and cell signaling (Table 4). Network analysis revealed that interacting genes were associated with lipid metabolism, small molecule biochemistry, cell-to-cell signaling and interaction and cellular development, cellular growth and proliferation, connective tissue development and function (Table 5).

**Table 4:** Ingenuity pathway analysis for sexually dimorphic genes: Biological functions under HFD conditions.

Category and Functions Annotation^a^	*p-value*	nb gene	regulationz-score	Molecules
**Cell Cycle**	**1,17E-04-4,68E-02**	**7**		
**Embryonic Development**	**1,28E-04-4,24E-02**	**19**		
development of organ	1,28E-04	18	1,439	ADAMTS1^f^, ADRA1A f, ALDH1A3^m^, C2orf65^f^, CACNB4^f^, CDSN^m^, CLDN1^f^, CST6 ^m^, CXADR^f^, Ddx3y ^m^, Eif2s3y ^m^, HCCS^m^, KDM5D ^m^, NCAM1^m^, OGT f, PSEN2^f^, TGFA^f^, TRIO^m^
				
**Organ Development**	**1,28E-04-4,24E-02**	**18**		
development of organ	1,28E-04	18	1,439	ADAMTS1^f^, ADRA1A f, ALDH1A3^m^, C2orf65^f^, CACNB4^f^, CDSN^m^, CLDN1^f^, CST6 ^m^, CXADR^f^, Ddx3y ^m^, Eif2s3y ^m^, HCCS^m^, KDM5D ^m^, NCAM1^m^, OGT f, PSEN2^f^, TGFA^f^, TRIO^m^
**Organismal Development**	**1,28E-04-4,24E-02**	**20**		
development of organ	1,28E-04	18	1,439	ADAMTS1^f^, ADRA1A f, ALDH1A3^m^, C2orf65^f^, CACNB4^f^, CDSN^m^, CLDN1^f^, CST6 ^m^, CXADR^f^, Ddx3y ^m^, Eif2s3y ^m^, HCCS^m^, KDM5D ^m^, NCAM1^m^, OGT, PSEN2, TGFA, TRIO^m^
**Tissue Development**	**1,28E-04-4,3E-02**	**26**		
development of organ	1,28E-04	18	1,439	ADAMTS1^f^, ADRA1A f, ALDH1A3^m^, C2orf65^f^, CACNB4^f^, CDSN^m^, CLDN1^f^, CST6 ^m^, CXADR^f^, Ddx3y ^m^, Eif2s3y ^m^, HCCS^m^, KDM5D ^m^, NCAM1^m^, OGT, PSEN2, TGFA, TRIO^m^
tissue development	4,04E-04	22	2,095	ADAMTS1^f^, ADRA1A f, ALDH1A3^m^, C2orf65^f^, CACNB4^f^, CDSN^m^, CLDN1^m^, CST6 ^m^, CXADR^f^, Ddx3y ^m^, Eif2s3y ^m^, HCCS^m^, KDM5D ^m^, LRRC17^m^, NCAM1^m^, OGT f, PPP2R2C ^m^, PSEN2^f^, PTN^m^, TGFA^f^, TRIO^m^, VAMP7^f^
aggregation of cells	2,69E-02	4	−0,300	CDSN^m^, FPR1 f, NCAM1^m^, SEPT5^m^
**Cellular Compromise**	**1,56E-04-1,58E-02**	**8**		
**Cellular Assembly and** **Organization**	**2,3E-04-4,68E-02**	**14**		
outgrowth of neurites	9,84E-03	5	1,929	NCAM1^m^, PPP2R2C ^m^, PTN,^m^ TRIO^m^, VAMP7^f^
**Cellular Development**	**2,3E-04-4,92E-02**	**14**		
growth of tumor cell lines	4,29E-02	7	1,065	CST6 ^m^, CXADR^f^, PPP2R2C ^m^, PTN^m^, SFRP4^f^, TGFA^f^, TXNIP
**Nervous System Development** **and Function**	**2,3E-04-4,43E-02**	**22**		
outgrowth of neurites	9,84E-03	5	1,929	NCAM1^m^, PPP2R2C ^m^, PTN^m^, TRIO^m^, VAMP7^f^
**Cell Signaling**	**2,33E-04-4,68E-02**	**9**		
quantity of Ca2+	7,24E-03	6	0,988	ADRA1A f, CORT^f^, FPR1 f, NCAM1^m^, PSEN2^f^, TGFA^f^
activation of blood cells	4,14E-02	6	0,142	BID^f^, Cd55/Daf2^f^, FPR1 f, PSEN2^f^, PSME2^m^, TXNIP^f^
activation of cells	4,91E-02	7	-0,289	BID^f^, Cd55/Daf2^f^, FPR1 f, PSEN2^f^, PSME2^m^, TGFA^f^, TXNIP^f^

a The first 10 biological categories of function are indicated and function annotation with a significant z-score are detailed.

f Genes more expressed in females. ^m^Genes more expressed in males.

Underline corresponds to genes both dimorphic under CD or HFD conditions.

**Table 5:** Ingenuity pathway analysis for sexually dimorphic genes: Networks under HFD conditions (M HFD vs F HFD).

ID		Score^a^	Top Functions/Molecules in Network^b^	
1	44		**Lipid Metabolism, Small Molecule Biochemistry, Cell-To-Cell Signaling and Interaction**	
			26s Proteasome, **ADAMTS1** f **,** **ADRA1A** f **,** Akt, **ALDH1A3** ^ m^ **,** **B3GAT1** f **,** **BID** f **,** **CACNB4** f **,** CD3, **Cd55/Daf2** f, Ck2, **CORT** f **,** **DZIP1** ^ m^ **,** ERK, ERK1/2, Focal adhesion kinase, **FPR1** f **,** **HCCS** ^ m^ **,** IL1, **KDM5D** ^ m^ **,** Mapk, **NCAM1** ^ m^ **,** NFkB (complex), **OGT** f, P38 MAPK, PI3K (complex), **PPP2R2C** ^ m^ **,** **PSEN2** ^ m^ **,** **PSME2** ^ m^ **,** **PTN** ^ m^ **,** **Retnlb** f **,** **SETD1A** ^ m^ **,** **TGFA** f **,** **TXNIP** f **,** Vegf	
2	25		**Cellular Development, Cellular Growth and Proliferation, Connective Tissue Development and Function**	
			**ALDH1A3** ^ m^ **, BHMT** ^ m^ **, CEP250** ^ m^ **, CHSY3** ^ m^ **,** FOS, FRZB, GJB6, HK3, **HPGDS** f **, HS3ST1** f **,** IFNB1, L3MBTL2, LIN9, **LIN37** ^ m^ **,** LIN52, LIN54, LMNA, **LRRC17** ^ m^ **,** **MAGEB18** ^ m^ **,** MYBL1, NCAPD3, NEK2, PEMT, PLXNB2, **PRPS1L1** f **, PRSS23** f **,** RECQL4, S100A7, TERF2, TNF, TP53, TPP1, tretinoin, **TRIO** ^ m^ **,** **VAMP7** f	

a Networks with scores ≥25 are indicated. ^b^Genes in bold are included in our datasets.

f Genes more expressed in females. ^m^Genes more expressed in males.

Underline corresponds to genes both dimorphic under CD or HFD conditions.

Thus, among the genes mapped in IPA, 97 genes were sexually dimorphic in CD conditions and 93 genes in HFD conditions. Interestingly, as shown on the Venn diagram (Figure 2A), 86 genes were dimorphic in basal conditions (CD) but not in HFD placentas, 81 were not dimorphic in basal conditions but became dimorphic under HFD conditions and only 11 genes displayed sex dimorphic expression in both CD and HFD placentas.

Among these 11 genes, *Adra1a, Eif2s3x, Kdm5c (Jarid1c)* and *Ogt* were more strongly expressed in female placentas (the last three are located on the X-chromosome) and *Cst6, Ppp2r2c, Zfp36l2, Ddx3y, Eif2s3y and Kdm5d (Jarid1d)* were more strongly expressed in male placentas. Interestingly, 4 Y-linked genes are represented on the microarray: *Ddx3y, Eif2s3y, Kdm5d* and Sry. The expression of this last gene is undetectable. Therefore we can observe male-specific expression for 3 out of 4 Y-linked gene in our microarray data. Notably, the amplitude of the differences in gene expression levels observed between males and females was very close under a CD as under a HFD. One gene, *Fpr1*, was more expressed in male CD-fed mother placentas and more expressed in female under HFD. However, the expression level was not sufficient for RT-qPCR validation.

Interestingly the expression of none of the known classical sex hormone receptor genes was sexually dimorphic under the constraint for IPA of *p*<0.01. Notably, however, for *p*<0.05, the *Estrogen Receptor Related beta (Esrrb)* or Nr3b2 and the hormone nuclear receptor *Retinoidx receptor gamma (Rxrg)* or Nr2b3 genes only were sexually dimorphic under a CD (more expressed in male, *p* = 0.030, and more expressed in female, *p* = 0.033, respectively).

#### The transcriptional response to maternal HFD is female- and male-specific

Given this striking sexual dimorphism, in order to characterize the effect of the maternal HFD on gene expression, and whether a HFD triggers different changes in males and females, we analyzed this effect in the two sexes separately. We compared the female HFD with the female CD microarrays and the male HFD with the male CD microarrays. We found that 168 genes were affected by maternal HFD in female placentas, with 164 genes mapped in IPA, whereas 190 genes were affected in male placentas, with 187 mapped in IPA. About half of these genes were upregulated, and half downregulated, in both female and male placentas (Table 1). The fold change (FC) distribution was equivalent in female and male, with most of the FCs included between −2.2 to −1.15 and 1.13 to 2.3. Interestingly, only 16 genes were dysregulated in both sexes, with the same amplitude (Figure 2B), namely *CCdC56, Sh3bgrl3 and Sumo1*, which were upregulated and *Cxcl2, GcGr, Klk8, Mpg, Pcca, Pdgfß, Pla2g15, Ppp2r3c, Rhbdf2, Slc7a2, Tmem62* and *Zfp37,* which were downregulated. Notably, *Fpr1* showed again as for sexual dimorphism a divergent regulation: it was upregulated in female and downregulated in male under a HFD.

According to the IPA analysis, the first 10 biological functions associated with the dysregulated genes in female placentas were involved principally in cell death, leukocytes stimulation and binding, amino acid metabolism, development and morphology of organ (digestive system, connective tissue) (Table 6). The four networks dysregulated in females were associated with, first, cellular development and hematopoietic system, second, cellular growth and proliferation, renal system development, third, free radical scavenging, cancer, cellular assembly and organization and last cell-to-cell signaling and interaction, cellular growth and proliferation, free radical scavenging (Table 7).

**Table 6:** Ingenuity pathway analysis for gene responding to HFD: Biological functions in female.

Category and Functions Annotation^a^	p-value	nb gene	regulationz-score	Molecules
**Cell Death**	1,09E-04-1,59E-02	18		
cell death of neuroblastomacell lines	0,00483	5	1,48	ATXN1^u^, MAOA^d^, SNCA^u^, TP53^u^, TXN^d^
**Cell Signaling**	1,09E-04-1,43E-02	7		
stimulation of lymphocytes	0,00490	4	0,337	C3^u^, ENPP3^u^, TNFSF15^d^, TXLNA^d^
binding of phagocytes	0,00626	4	0,082	C3^u^, CXCL2 ^d^, FPR1 u, LIPG^d^
quantity of monoamines	0,00786	4	1,978	C3^u^, MAOA^d^, SLC22A3, SNCA^u^
stimulation of cells	0,00892	6	0,297	C3^u^, CCK^u^, ENPP3^u^, EPX^d^, TNFSF15^d^, TXLNA^d^
**Cellular Development**	2,92E-04-1,4E-02	11		
growth of tumor cells	0,00957	5	−0,119	COX17^u^, PDGFB ^d^, TFG^u^, TP53^u^, TXLNA^d^
**Digestive System Development** **and Function**	2,92E-04-7,02E-03	5		
**Organ Development**	2,92E-04-1,4E-02	3		
**Organ Morphology**	2,92E-04-1,4E-02	6		
**Small Molecule Biochemistry**	2,92E-04-1,43E-02	23		
uptake of amino acids	0,00042	4	−0,873	SLC1A1^d^, SLC36A1^u^, SLC7A2 ^d^, TP53^u^
quantity of glutathione	0,00084	4	1,789	SNCA^u^, TP53^u^, TXN^d^, VNN1^d^
phosphorylation of L-tyrosine	0,00154	5	0,410	CCK^u^, FGF10^d^, GATA1^d^, PDGFB ^d^, TP53^u^
quantity of monoamines	0,00786	4	1,978	C3^u^, MAOA^d^, SLC22A3, SNCA^u^
**Tissue Development**	2,92E-04-1,4E-02	11		
mass of connective tissue	0,01490	4	−0,842	C3^u^, GCGR ^d^, RXRG^d^, TP53^u^
**Amino Acid Metabolism**	4,2E-04-1,4E-02	9		
uptake of amino acids	0,00042	4	−0,873	SLC1A1^d^, SLC36A1^u^, SLC7A2 ^d^, TP53^u^
phosphorylation of L-tyrosine	0,00154	5	0,410	CCK^u^, FGF10^d^, GATA1^d^, PDGFB ^d^, TP53^u^
**Molecular Transport**	4,2E-04-1,4E-02	16		
uptake of amino acids	0,00042	4	−0,873	SLC1A1^d^, SLC36A1^u^, SLC7A2 ^d^, TP53^u^
quantity of glutathione	0,00084	4	1,789	SNCA^u^, TP53^u^, TXN^d^, VNN1^d^
quantity of monoamines	0,00786	4	1,978	C3^u^, MAOA^d^, SLC22A3, SNCA^u^

a The first 10 biological categories of function are indicated and function annotation with a significant z-score are detailed.

u Upregulated genes. ^d^Downregulated genes.

Underline corresponds to genes deregulated both in female and male placentas.

**Table 7:** Ingenuity pathway analysis for gene responding to HFD: Networks in female.

ID	Score^a^	Top Functions/Molecules in Network^b^
1	38	**Cellular Development, Hematological System Development and Function, Hematopoiesis**
		14-3-3, 20s proteasome, 26s Proteasome, Alpha tubulin, **ATP5F1** u, **ATXN1** u, **CDT1** ^d^ **,** **DEK** ^d^ **,** Elastase, **EPN2** ^d^ **,** **GATA1** ^d^ **,** Hat, HISTONE, Hsp27, Hsp70, IFN Beta, Ikb, IL1, MARK3, **MMP1** u, **NDUFA5** u, NFkB (complex), Nos, **PNMA1** ^d^ **, PRMT7** ^d^ **, RGL2** ^d^ **,** **RIT1** u, **SNCA** u, **TCEANC** ^d^ **,** **TFG** u **,** **TNFSF15** ^d^ **,** **TPMT** u **,** **TPSAB1/TPSB2** ^d^ **, TXN** ^d^, Ubiquitin
2	35	**Cellular Growth and Proliferation, Renal Proliferation, Renal and Urological System Development and Function**
		**AGA** u **,** Alp, **ATRIP** ^d^ **,** **C3** u **,** **CCK** u **,** Collagen type I, Collagen(s), Creb, **CTH** ^d^ **,** **CXCL2** ^d^ **,** Cyclin A, ERK1/2, **FGF10** ^d^ **,** HDL, **HS3ST1** u **,** **KCNA5** ^d^ **,** LDL, **LIPG** ^d^ **,** N-cor, Pdgf (complex), PDGF BB, **PDGFB** ^d^ **,** PLA2, PP2A, **PPAP2C** u **,** Rsk, **RXRG** ^d^ **,** **SLC1A1** ^d^ **,** sulfotransferase, **SULT1E1** u **,** **SULT2B1** ^d^ **,** **TEAD4** ^d^ **,** Tgf beta, **VNN1** ^d^ **, WWTR1** ^d^
3	28	**Free Radical Scavenging, Cancer, Cellular Assembly and Organization**
		**AACS** ^d^ **,** **AGTR1** ^d^ **,** BAI3, Caspase, Ck2, **COX17** u, **DAP** u **,** **EFTUD2** ^d^ **,** FFAR1, FFAR3, Focal adhesion kinase, **GCGR** ^d^ **,** **GCM1** ^d^ **,** Gpcr, **GPR4** ^d^ **,** GPR77, GPR87, **GPR152** u **,** Histone h3, **HPGDS** u **,** Insulin, Jnk, **KLC1** ^d^ **,** **MAOA** ^d^ **,** Mapk, PI3K (complex), Pkc(s), **PLB1** ^d^, PLC, **PRELID1** u **,** Rac, Ras, **RPS27L** u **,** Sos, **TP53** u
4	25	**Cell-To-Cell Signaling and Interaction, Cellular Growth and Proliferation, Free Radical Scavenging**
		**ACP5** u **,** Akt, Ap1, **ATPIF1** u **, CAMK2N1** u **,** **CYTIP** ^d^ **,** ERK, Estrogen Receptor, **FPR1** u **,** FSH, hCG, **IGDCC3** ^d^ **,** IgG, IL12 (complex), **IL3RA** ^d^ **,** Immunoglobulin, Integrin, Interferon alpha, **KLK7** ^d^ **, KLK8** ^d^, Lh, **MED29** ^d^ **,** Mediator, Nfat (family), **NID2** ^d^ **,** P38 MAPK, p85 (pik3r), Pka, RNA polymerase II, **RPL23A** ^d^ **,** **SOCS4** u **,** **Sumo1** u **,** **TXLNA** ^d^ **,** **TYRO3** ^d^ **,** Vegf

a Networks with scores ≥25 are indicated. ^b^Genes in bold are included in our datasets.

u Upregulated genes. ^d^Downregulated genes.

Underline corresponds to genes deregulated both in female and male placentas.

In male placentas, the first 10 biological functions associated with the dysregulated genes were principally involved in development and function of cardiovascular system (development of vessel, pressure), metabolism of fatty acid (oxidation and quantity) and glucose (uptake, quatity), extansion of protrusions and development of nervous system (Table 8). The 6 networks of genes dysregulated in male placentas corresponded to, first, cellular growth and proliferation, connective tissue development, cellular movement, second, cellular movement, hematological system, immune cell trafficking, third, connective tissue, genetic and dermatological disorders, fourth, cell death, cellular development, cellular growth and proliferation, fifth, cell death, gene expression cellular growth and proliferation and last cellular development, cell cycle, connective tissue development and function (Table 9).

**Table 8:** Ingenuity pathway analysis for gene responding to HFD: Biological functions in male.

Category and Functions Annotation a	p-value	nbgene	regulationz-score	Molecules
**Cardiovascular System Development** **and Function**	1,88E-06-1,1E-02	24		
blood pressure	0,00000	11	−1,989	ACTG2^u^, ADRA2A^u^, AOC3^u^, CACNA1B^d^, COL1A2^u^, CYP4A11^d^, GCGR ^d^, HSD11B1^d^, IGF1^u^, SGK1^d^, SNTB2^d^
development of blood vessel	0,00087	14	0,104	ADRA2A^u^, AOC3^u^, COL1A1^u^, COL1A2^u^, COL3A1^u^, CXCL2 ^d^, GJA1^d^, IGF1^u^, IRS1^d^, LTB4R^d^, PDGFB ^d^, PITX2^u^, SFRP2^u^, TBX3^d^
systolic pressure	0,00129	4	−1,971	ADRA2A^u^, CYP4A11^d^, IGF1^u^, SNTB2^d^
**Embryonic Development**	1,43E-05-1,1E-02	30		
development of organ	0,00125	26	0,041	ADRA2A^u^, CD14^d^, CEBPA^d^, CELSR1^u^, COL1A1^u^, COL1A2^u^, COL3A1^u^, CRABP2^d^, GJA1^d^, HAND2^d^, HSD11B1^d^, IGF1^u^, IRS1^d^, MAB21L2^u^, MAS1^d^, PDGFB ^d^, PITX2^u^, PLAG1^u^, RAB3A^u^, RAX^d^, ROR2^u^, SFRP2^u^, TBX3^d^, VCAN^d^, WFDC2^d^, ZFP37 ^d^
**Organismal Development**	1,43E-05-1,1E-02	34		
development of blood vessel	0,00087	14	0,104	ADRA2A^u^, AOC3^u^, COL1A1^u^, COL1A2^u^, COL3A1^u^, CXCL2 ^d^, GJA1^d^, IGF1^u^, IRS1^d^, LTB4R^d^, PDGFB ^d^, PITX2^u^, SFRP2^u^, TBX3^d^
development of organ	0,00125	26	0,041	ADRA2A^u^, CD14^d^, CEBPA^d^, CELSR1^u^, COL1A1^u^, COL1A2^u^, COL3A1^u^, CRABP2^d^, GJA1^d^, HAND2^d^, HSD11B1^d^, IGF1^u^, IRS1^d^, MAB21L2^u^, MAS1^d^, PDGFB ^d^, PITX2^u^, PLAG1^u^, RAB3A^u^, RAX^d^, ROR2^u^, SFRP2^u^, TBX3^d^, VCAN^d^, WFDC2^d^, ZFP37 ^d^
**Lipid Metabolism**	1,96E-05-1,18E-02	18		
oxidation of fatty acid	0,00454	5	−0,080	CYP3A4^u^, CYP4A11^d^, HADH^u^, HSD11B1^d^, IRS1^d^
quantity of triacylglycerol	0,01100	5	−1,845	APOA5^d^, BHMT^d^, CEBPA^d^, HSD11B1^d^, IGF1^d^
**Small Molecule Biochemistry**	1,96E-05-1,18E-02	27		
uptake of 2-deoxyglucose	0,00410	4	−0,790	CEBPA^d^, FPR1 ^d^, IGF1^u^, IRS1^d^
oxidation of fatty acid	0,00454	5	−0,080	CYP3A4^u^, CYP4A11^d^, HADH^u^, HSD11B1^d^, IRS1^d^
quantity of D-glucose	0,00562	5	0,217	ADRA2A^u^, GCGR ^d^, HADH^u^, IGF1^u^, IRS1^d^
quantity of triacylglycerol	0,01100	5	−1,845	APOA5^d^, BHMT^d^, CEBPA^d^, HSD11B1^d^, IGF1^d^
**Cell Morphology**	2,6E-05-9,22E-03	9		
extension of cellular protrusions	0,00922	5	−0,202	ARHGAP4^u^, IGF1^u^, LTB4R^d^, ROR2^u^, S100B^u^
**Nervous System Development** **and Function**	2,6E-05-1,1E-02	34		
memory	0,00023	7	0,563	GABRB3^u^, GJA1^d^, HSD11B1^d^, IGF1^u^, KLK8, S100B^u^, SGK1^d^
spatial memory	0,00050	4	0,217	GJA1^d^, HSD11B1^d^, IGF1^u^, S100B^u^
proliferation of neuroglia	0,00064	5	−0,06	CXCL2 ^d^, IGF1^u^, PDGFB ^d^, PLAG1^u^, S100B^u^

a The first 10 biological categories of function are indicated and function annotation with a significant z-score are detailed.

u Upregulated genes. ^d^ Downregulated genes.

Underline corresponds to genes deregulated both in female and male placentas.

**Table 9:** Ingenuity pathway analysis for gene responding to HFD: Networks in male.

ID		Score^a^	Top Functions/Molecules in Network^b^
1	43		**Cellular Growth and Proliferation, Connective Tissue Development and Function, Cellular Movement**
			**ACTG2** u **,** **ADD3** ^d^ **,** Alp, **CDH11** u **,** **CEACAM3** ^d^ **,** Collagen Alpha1, Collagen(s), **CRABP2** ^d^ **,** ENaC, ERK1/2, **GDA** ^d^ **,** **GJA1** ^d^ **, GKN1** u **, IGF1** u **, IGSF1** u **,** **IRS1** ^d^ **,** **KLRD1** ^d^ **,** Laminin, **MTUS1** ^d^ **,** **P4HA3** u **,** Pdgf (complex), PDGF BB, **PDGFB** ^d^ **,** PP2A, Rock, **ROR2** u **, S100B** u **, SCNN1G** ^d^ **,** **SGK1** ^d^ **,** Sos, **TAGLN** u **,** **TBX3** ^d^ **,** Tgf beta, **TRIOBP** u **,** Trypsin
2	38		**Cellular Movement, Hematological System Development and Function, Immune Cell Trafficking**
			**4930486L24Rik** ^d^ **,** **Actg2** u **,** ADCY, **ADRA2A** u **, AOC3** u **, APOA5** u **, CELSR1** u **, CMKLR1** u **, CXCL2** ^d^ **,** **ECT2** ^d^ **,** F Actin, **FPR1** ^d^ **,** G protein, **GCGR** ^d^ **,** Gi-coupled receptor, Gpcr, Gsk3, **HADH** u **, HLA-DRB1** ^d^ **,** IL12 (family), Immunoglobulin, Insulin, **LTB4R** ^d^ **,** **MAS1** ^d^ **, P2RY1** ^d^ **,** p85 (pik3r), PI3K (complex), PLC, Proinsulin, **RAB3A** u **,** **RAMP3** ^d^ **,** Ras homolog, **RHPN1** u **,** Shc, **SNX33** ^d^
3	35		**Connective Tissue Disorders, Genetic Disorder, Dermatological Diseases and Conditions**
			Alpha catenin, C/ebp, **CASP4** ^d^ **, CD14** ^d^ **, CLCF1** u **, COL1A1** u **, COL1A2** u **, COL3A1** u **, COL4A5** u **, COL6A1** u **, COL6A2** u **, COL6A3** u **,** collagen, Collagen type I, Collagen type III, Collagen type IV, **CYP3A4** u **,** **CYP4A11** ^d^ **,** Growth hormone, **HOXD10** ^d^ **,** **HSD11B1** ^d^ **,** Hsp27, Ifn, IFNK, Iga, IL1, JAK, **MMP16** u **,** NFkB (complex), **PCOLCE** u **,** Pdi, Tlr, Tnf, **TNFSF8** ^d^ **,** **VCAN** u
4	27		**Cell Death, Cellular Development, Cellular Growth and Proliferation**
			Akt, Ap1, **ASF1B** u **,** Caspase, **CCDC80** u **,** **CEBPA** ^d^ **,** E2f, **GLRX2** u **,** **GSTM1** ^d^ **,** **HAND2** ^d^ **,** hCG, Hdac, **HIST1H3A** u, Histone h3, Histone h4, Hsp70, Hsp90, Ige, IgG, Igm, Interferon alpha, **KLK8** ^d^ **,** MAX, Mek, Nfat (family), P38 MAPK, **PITX2** u **, PLAG1** u **, SFRP2** u **,** **SLA** ^d^ **,** **ST6GAL1** u **, Sumo1** u **, SUV39H2** ^d^ **, TRAF1** ^d^ **,** Wnt
5	26		**Cell Death, Gene Expression, Cellular Growth and Proliferation**
			ADAMTS5, **ARHGAP4** u **,** beta-estradiol, BPIFA1, **CCDC80** u **,** CD300C, **CRABP2** ^d^ **,** CRADD, DLEU1, E2F1, ERBB2, FMO1 (includes EG:14261), HDGF, HEBP1, **INMT** ^d^ **,** KCNQ5, **LOXL2** u **,** mir-26, **ODZ3** u **, OLFML2B** u **,** **PLA2G15** ^d^ **,** **SH3BGRL3** u **, SLC14A1** u **,** **SLC7A2** ^d^ **,** SMAD4, SMPDL3A, **SP2** u **,** SPARCL1, **SPRR3** ^d^ **,** ST3GAL4, TNF, **TNFSF8** ^d^ **,** tretinoin, UCK1, **WFDC2** ^d^
6	26		**Cellular Development, Cell Cycle, Connective Tissue Development and Function**
			**B630019K06Rik** u **,** CDK4, CNP, COL7A1, DBN1, DLG4, **EMILIN3** u **,** EPC2, **ERAS** u **,** **FAM135B** ^d^ **,** **GPA33** u **,** GPER, KAT5, KIF1A, KLF4, LGI1, **LRFN2** u **,** MAPK1, Max-Myc, MAZ, **MPG** ^d^ **,** MYC, OAS1, **PGAP1** u **, PSME2** u **, RBMS3** u **,** **RHBDF2** ^d^ **,** SERINC3, SP1, **SPRR3** ^d^ **,** SQSTM1, **TBX3** ^d^ **, THNSL1** ^d^, TNNT3, **VMAC** u

a Networks with scores ≥25 are indicated. ^b^Genes in bold are included in our datasets.

u Upregulated genes. ^d^Downregulated genes.

Underline corresponds to genes deregulated both in female and male placentas.

Among dysregulated genes and gene families, some have been reported to be important for placental development [36], [37], [38]: Adrenergic receptor *Adra2a*, *Ceacam10*, *Crabp2, Gata1*, *Gcm1,* Matrix metalloproteases (*Mmp1a and 16*), *Pdgfß* and *Rarb*.

### Epigenetic Analyses

#### Effect of maternal diet and sexual dimorphism on the epigenetic machinery enzymes

As there is increasing evidence that epigenetic mechanisms are involved in both DOHaD and sexual dimorphism [39], we checked whether genes encoding enzymes involved in the epigenetic machinery displayed changes in expression in our microarrays. Here, the *p*-value threshold was considered as *p*≤0.05. Seven genes were differentially expressed in our analysis: the genes encoding the DNA methyltransferase 3 cofactor, *Dnmt3l*, the H3K9 trimethyltransferases, *Kmt1a* and *Kmt1b* (*Suv39h1* and *Suv39h2)*, the protein arginine methyltransferase *Prmt7,* the H3K4 methyltransferase *Kmt2f (Setd1a)* and the H3K4 demethylases, *Kdm5c* and *Kdm5d* (Table 10). *Dnmt3l, Kmt1a* and *Kmt2f* were downregulated in the F HFD placentas (in comparisons with F CD placentas); a similar trend for downregulation was observed in male placentas (M HFD *vs* M CD) but not statistically significant. *Kmt1b* was downregulated in M HFD placentas with respect to M CD placentas; a similar trend for downregulation was observed in female placentas (F HFD *vs* F CD) but not statistically significant. *Prmt7* was downregulated in HFD group in both sexes. Moreover, sexual dimorphism was observed for *Kdm5c* and *Kdm5d*, under CD and HFD maternal diet. Given that *Kdm1d* is on the Y chromosome it is therefore only expressed in males. *Kmt2f* was more strongly expressed in male than in female placentas in HFD conditions. Effects were significantly validated in RT-qPCR analysis, except for *Prmt7* (non significant) and *Kmt2f* (not done, Supporting figure S2). The expression of *Dnmt3a, Dnmt3b* and *Dnmt1* did not differ between the sexes or between diets. Thus, the expression of *Dnmt3l*, *Kmt1a, Kmt1b, Kmt2f and Prmt7* was affected by maternal HFD and the expression of *Kdm5c* and *Kdm5d* showed higher levels of expression in female and male placentas respectively, and confirmed the robutsness of microarray analysis.

**Table 10:** Effect of sex and maternal diet on the epigenetic machinery enzyme gene expression.

Gene name	Enzyme function	Sexual dimorphism:FCD vs MCD	Sexual dimorphism: FHFD vs MHFD	Response to Maternal diet: FHFD vs FCD	Response to Maternal diet: MHFD vs MCD	Level of associated mark
***Dnmt3l***	Dnmt3 cofactor, DNA methylation			? (−0.38/−0.53)		hypomethylation in F HFD vs F CD
***Kmt1a (Suv39h1)***	H3K9 methyltransferase			? (−0.43/−0.66)	? (NS/−0.58)	no difference in H3K9me3 level.
***Kmt1b (Suv39h2)***	H3K9 methyltransferase	M>F (−0.45/NS)			?	no difference in H3K9me3 level.
***Kdm5c (Jarid1c)***	H3K4 demethylase	F>M (0.36/0.55)	F>M (0.30/0.48)			no difference in H3K4me3 level.
***Kdm5d (Jarid1d)***	H3K4 demethylase	M>F (−2.36/−6.26)	M>F (−2.42/−6.37)			no difference in H3K4me3 level.
***Kmt2f (Setd1a)***	H3K4 methyltransferase		M>F (−0.22/ND)	? (−0.15/ND)		no difference in H3K4me3 level.
***Prmt7***	H3R2 methyltransferase			? (−0.27/NS)	? (−0.25/NS)	not done (no antiH3R2me1 Ab)

Log_2_(fold change) are indicated in brackets (microarrays/RT-qPCR). ND = not done. NS = non significant.

#### Investigation of the effects of maternal diet and sexual dimorphism on relevant histone marks

**Figure 3:**
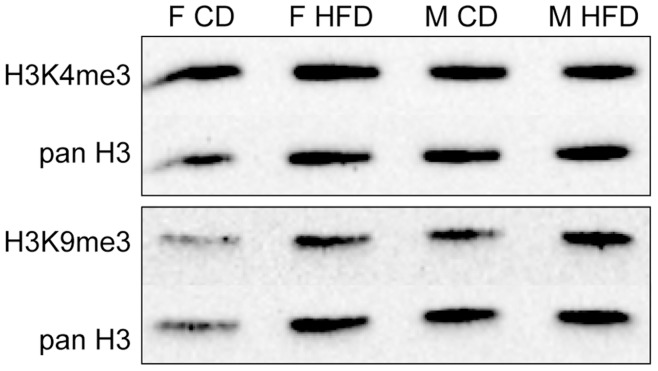
Western blotting with antibodies specific for H3K4me3 and H3K9me3. was used to determine the degree of lysine-specific methylation, in relationship with Kdm5c/5d (H3K4me3 demethylase) and Kmt1a/1b (H3K9 trimethylases). No sex- or diet-specific signal was observed.

Since Kdm5c, Kdm5d, Kmt1a and Kmt1b enzymes are responsible for histone modifications of H3K4 and H3K9, respectively, we investigated whether the differences in gene expression led to differences in the global level of pertinent histone methylation marks. We extracted histones and performed western blotting on the placenta samples, probed with anti-H3K4me3 or anti-H3K9me3 antibodies, and with anti-panH3 antibody as a control (Figure 3). We did not detect any changes in the global level of these two marks between the four different groups (*p* = 0,6022 for H3K9me3; *p* = 0,9990 for H3K4me3, Kruskal-Wallis non-parametric test, n = 3–4 technical replicates). As no antibody for H3R2me1 was available, we were not able to study this mark brought by Prmt7.

## Discussion

Evidence for non genetic inheritance is accumulating, with marked sexual dimorphism observed in some cases, both for the mode of transmission and the resulting effects [40]. There is accumulating evidence for the role of the placenta as a programming agent [8]. However while transcriptomic analysis are emerging [17], [41] epigenetic bases in connection with nutrition are less documented [29], [42]. Thus, to our knowledge, this study is the first to report sex-specific functional differences based on both epigenetic and transcriptomic analyses related to diet response in the mouse placenta. Transcriptomic data revealed sexually dimorphic placental gene expression not only under a HFD but also under CD. The effect of a maternal HFD on placental gene expression was thus clearly sexually dimorphic. As shown here males and females diverge not only in terms of number and variation of genes but more specifically in the functions and networks involved; mainly in cell signalling involving immune cells and uptake and metabolism of aminoacids for females and development and function of vascular system and uptake and metabolism of glucose and fatty acids for males. This study also brings new insight into implication of the epigenetic machinery in placental programming. There was thus conspicuous sexual dimorphism for programming trajectories in response to the same environmental challenge, a maternal HFD.

### Neither Sex Nor Maternal HFD Trigger Gross Morphological Changes in Placental Layers

Experimental and epidemiological studies in humans and animal models have demonstrated that predisposition to impaired glucose tolerance, blood pressure and coronary heart disease are associated with either low or high fetus-to-placenta weight ratio index (FPI) [43]. We previously showed an increase in placental weight and a decrease in FPI under maternal HFD, for both male and female placentas [29]. This indicates a change in placental efficiency, which could be due to either a change in placental development or to gross morphological changes in the differents layers. Using in situ hybridization with several probes, we could not detect any changes in the respective proportion of the two layers [29]. In the present study, our histological analysis identified no differences in the labyrinth exchange zone between the four groups, in terms of area and proportion but, as shown by the dysregulation of important genes involved in placental development [36], [38], we cannot exclude subtle changes. Athogether this suggests that the observed increase in placental weight was distributed evenly between the layers. These changes were observed at E15.5, but it would be of interest to investigate the changes at term. The impaired development of the placenta in conditions of maternal overnutrition is consistent with the role of the placenta in programming and subsequent impaired responses in adulthood [8], [9], [15], [17], [43], [44], [45], [46]. Like most expression studies in mice, we analyzed whole placentas that contain mixed cell populations [17], [18], [47]. Changes in the proportions of the different cell populations might generate the differences observed, independently of changes in DNA methylation or gene expression in a single cell population. Although we cannot exclude the possibility of modifications to placental structure below the level of detection of the techniques used.

### Gene Expression both at Basal Level and in Response to Maternal HFD is Sexually Dimorphic

Several studies have reported differences in gene expression between male and female placentas in humans and mice [17], [24], [25], [48]. In humans, Clifton *et al.* showed that the placenta adapts in a sexually dimorphic manner to chronic maternal asthma. The growth of female fetuses is reduced, increasing chances of survival, whereas a normal growth of male fetuses is associated with a poor outcome in the case of acute asthma exacerbation [21], [48]. In mice, Mao et al reported that in E12.5 mice fed a low-fat diet (10%) or a very high-fat diet (60%), similar to the CD and HFD used in this study, respectively, or an intermediate chow diet (25%), there were more changes in gene expression with both diets in female than male placentas [17]. In the present report, but at a later stage, E15.5, we confirmed that the affected gene sets differ between females and males. However our transcriptomic analysis provides no evidence for a greater reactivity to maternal HFD in either male or female mouse placentas, in terms of placental and fetal growth or the numbers of dysregulated genes. Moreover, using the Ingenuity Pathway Analysis, we show for the first time that the gene sets change not only from a quantitative point of view but also, more strikingly, from a qualitative point of view. Indeed, the associated biological functions affected by the HFD, differed markedly between the two sexes. In the absence of a greater reactivity but with clearly different trajectories, it is difficult to say whether one sex copes better than the other under a HFD, as previously suggested in other contexts [21], [49]. Differences in adaptation between males and females may therefore be context-, species- and stage-specific.

Under control conditions, the function and networks associated with sexually dimorphic genes were mainly associated with immune response and chemotaxis of immune cells. In placenta, these functions are associated with inflammatory response, fetal-maternal immune protection and are implicated in vascular development processes [50], [51]. The feto-placental ratio index being different between males and females [29], these processes could contribute in the lower efficiency of male placenta under control conditions.

Under maternal HFD, the sexually dimorphic functions and networks accounted for development of organ and tissue, growth of neurites and tumor. These functions are consistent with the functions associated with differentially expressed genes in male in response to maternal HFD. In males, function annotations are linked with blood pressure and development of blood vessels. Neurites and blood vessels are branching organs that share the same molecular base. Moreover, in males only, many collagene genes involved in connective tissue disorders, were downregulated, and these are implicated in development of blood vessels. Therefore, one dimorphic mechanism in response to maternal HFD may be vascular development, more affected in male than in female placenta. Several reports observed vascular abnormalities in response to maternal obesity, and HFD but none described differences between sexes [11], [52], [53].

Males and females show the involvement of “small molecule biochemistry” function. However in females this annotion is linked with uptake of amino acids and monoamines, while in males this function is linked with uptake of glucose and metabolism of fatty acids. This demonstrates clearly that the programming trajectories induced by diet in placenta are specific to each sex. Thus, the sexually dimorphic response of the fetus is derived in part from sex-linked differences in placental adaptation to a physiopathological condition. Interestingly Jones et al reported that HFD increased transplacental transport of glucose and neutral amino acids (system A) in mouse placenta at 18.5, but without discriminating between males and females [54], thus highlighting the importance of studying both sexes in dietary interventions.

Sexual dimorphism in placenta could result from differential effects of sex hormones and sex chromosomes. There was no difference in gene expression for any of the known classical sex hormone receptor genes, but only the genes encoding the Estrogen Receptor Related-beta Nr3b2 and the hormone nuclear Retinoidx receptor gamma Nr2b3 were sexually dimorphic. However the orphan nuclear receptor Nr3b2 activate gene promoters in a ligand-independent manner via interactions with Estrogen Response Elements sequences [55]. This result and the modest effect that the fetal sex hormones may have in placenta at this stage bring to the fore the role of sex chromosomes.

Indeed, sexual dimorphism was also observed in total embryonic cells taken from mice at E10.5, before sexual differentiation [56]. Remarkably, these cells responded differently to the applied dietary stressors, even before the production of fetal sex hormones. This difference in cell behavior and sensitivity appears to be driven by the genetic sex of the cells from the outset, with the effects of factors such as hormones subsequently being superimposed on this difference. In bovine blastocysts, sex determines the level of expression of one third of the actively expressed genes [57]. In human term placentas, Sood *et al.* showed that many of the sex-correlated genes were located on the sex chromosomes, but that some were autosomal [25]. In our study, we cannot conclude that there are more genes on the Y or X chromosome directly accounting for sexually dimorphic placental gene expression in basic conditions. We found that the X- and Y-linked genes, *Eif2s3x, Ogt* and *Kdm5c* on the X and *Eif2s3y, Ddx3y and Kdm5d* on the Y, are sexually dimorphic in our study, whatever the diet. Interestingly, these gene have also been reported as sexually dimorphic in other microarray analysis: *Ddx3y*, *Eif2s3y*, *Kdm5d* and *Ogt* are sexually dimorphic in E12.5 placenta [17]; Kdm5c was also more expressed in female in human placenta [25]; *Ddx3y*, *Eif2s3y* and *Kdm5d* are male-specific in mouse hearts and human myocardium [58].

Despite the occurrence of many X-homologous regions distributed along the Y-chromosome, most of these do not recombine and are designated as male-specific region, which contains 78 single and multicopy genes that encode about 27 distinct proteins in humans [59], [60]. In the pseudo-autosomal regions (PAR), 29 genes are conserved on X and Y chromosomes. In our study, neither of the X and Y linked genes that were differentially expressed are on the PAR. Two X-Y paralogues of sexually dimorphic gene (*Eif2s3x* and *Eif2s3y,* and *Kdm5c* and *Kdm5d)* are homologous. In another report, in mouse brain, the expression of the Y version in males for these two genes pairs did not compensate for the dosage imbalance between the two sexes in the expression of their X homologues escaping X-inactivation [61]. X- and Y-linked genes such as *Eif2s3x, Eif2s3y, Kdm5c Kdm5d, Ogt* and *Ddx3y* may modulate the expression of different sets of autosomal genes, leading to physiological differences between males and females [40].

### Epigenetic Changes

The mouse extraembryonic membranes, yolk sac and placenta are characterized by global undermethylation of DNA with respect to embryonic somatic lineages. The major differences in methylation between the two lineages probably affect non gene regions, such as, for example, centromeric heterochromatin [62]. Indeed, in both chorionic villi and placental fibroblasts, large differences have been observed both globally and between various chromosome structures within individual metaphases [63]. In a previous report, we used the LUMA technique to analyze DNA methylation: female placentas displayed significantly higher levels of methylation than male placentas under control conditions [29]. We also showed an effect of maternal HFD, with hypomethylation in female placentas only [29]. However in the present paper, the levels of expression of neither *Dnmt3a, Dnmt3b* nor *Dnmt1* differed between the sexes or between diets. In contrast, this hypomethylation was consistent with the observation of *Dnmt3l* downregulation only in female HFD placentas. *Dnmt3l* has no DNA methylase activity but is a cofactor of the *de novo* DNA methyltransferases [64], [65]. Our data do not exclude a role for *Dnmt3a, Dnmt3b* and *Dnmt1* in global methylation differences; indeed the expression of these genes and the levels of the corresponding proteins may have varied before the E15.5 stage. Time course studies and studies of the cell type distribution of DNA methylation during development are therefore of interest.

Six other epigenetic machinery genes, *Kmt1a* and *Kmt1b, Kmt2f, Kdm5c* and *Kdm5d*, and *Prmt7* were dysregulated under maternal HFD, as an effect of diet, sex of the fetus, or both. *Kmt1a* and *Kmt1b* encode methyltransferases involved in trimethylation of the lysine 9 residue of histone H3 (H3K9me3 is mostly associated with a repressed chromatin state). *Kdm5c* and *5d*, demethylases of lysine 4 of histone H3 (H3K4me3 is mostly associated with an active chromatin state), maps to the X and Y chromosomes respectively. *Kdm5c* escapes X-inactivation and is more strongly expressed in females than in males [66]. The two paralogues are highly similar in nucleotide and amino acid sequence but whether the role and targets of these 2 enzymes are identical, divergent or with partial compensation in their functions is still unclear [61]. *Prmt7* is an arginine methyl transferase (H3R2) and *Kmt2f* is a H3K4 methyltransferase. Little is known about these two enzymes and their role is not described in placenta.

The involvement of all these enzymes in an important network is consistent with: a) documented crosstalk between the H3K4 and H3K9 methylation marks, with H3K9 methylation partly controlled by the Kmt1a and 1b enzymes, and b) crosstalks between histone methylation and acetylation and DNA methylation. Dnmt3l has been shown to recruit histone deacetylases [67] and to interact with H3K4me3 [68]. Moreover, the H3K4 demethylase Kdm5c interacts with H3K9me3, which can be methylated by Kmt1a and 1b [69]. Kmt1a has recently been implicated in the memorization of hyperglycemia in the endothelial cells of diabetic patients, highlighting the key role of this enzyme of the epigenetic machinery in transducing the impact of the environment on metabolic gene expression [70]. It will therefore be important to determine how the crosstalk between key repressive or activating marks and their modifying enzymes can be disturbed by environmental challenges, leading to developmental and metabolic malprogramming.

In line with the changes in expression of histone modifying enzymes, we analyzed global histone modifications. The H3K4me3 signal was not sexually dimorphic, as might have been expected given the dimorphic expression of *Kdm5c* and *Kdm5d*, and was equivalent in both diet despite the dysregulation of *Kmt2f.* Similarly, H3K9me3 was not affected by diet despite the downregulation of *Kmt1a* and *Kmt1b.* However, western blotting provides a global evaluation of histone marks and cannot exclude subtle changes in particular target sequences of these enzymes. The identification of histone marks modifications at the level of the whole genome will therefore be of interest. Thus, diet itself or the mechanisms used by cells to compensate for dietary imbalances for adaptation must have a major impact on the epigenetic machinery. As previously highlighted for DNA methylation, time course studies and studies of the cell type distribution of histone marks during development are required.

These findings highlight the importance of studying both sexes in epidemiological protocols or dietary interventions, in both humans and experimental animal models. Our results pave the way for explorations concerning the possible targeting, by fatty acids and other nutrients, of conspicuous regions in the genome harboring binding sites for the recruitment of diet- and tissue-specific chromatin remodeling complexes. Elucidation of the ways in which epigenetic modifications fix the effects of early environmental events, in a sex-specific manner, ensuring sustained responses to transient stimuli resulting in modified gene expression patterns and phenotypes later in life, remains a key challenge [27].

## Supporting Information

Figure S1 **Analysis of E15.5 placental structure.** on HE-stained paraffin sections, of the placental layers in female and males (F or M) from mother fed a control (CD) or high-fat (HFD) diet. **(A)** Details of the labyrinth layer. **(B)** Details of the junctional zone. Scale bars indicate 200 µm. **(C)** Measurement of the area and shape of the labyrinth and total placenta on histological slides. “Minor” and “major” indicate the width and length. Data are presented in arbitrary units, as means ± SEM (n = 8 per group). Non-parametric Kruskal-Wallis statistical tests indicated an absence of difference between the four groups for all measurements.
(PDF)

Figure S2 **Histograms of epigenetic machinery expression levels in microarrays and RT-qPCR.** Line indicates statistical significance and the number refers to log_2_ fold change.
(PDF)

Table S1 **Limma tables for the 4 comparisons (F HFD vs CD, M HFD vs CD, CD F vs M, HFD F vs M).** Excel file.
(XLS)
