# Supplementary material for: Maternal Diets Trigger Sex-Specific Divergent Trajectories of Gene Expression and Epigenetic Systems in Mouse Placenta
Source: PLoS One. 2012 Nov 5;7(11):e47986. doi: 10.1371/journal.pone.0047986 (PMC3489896; doi:10.1371/journal.pone.0047986)
Supplement: Figure S1 — Analysis of E15.5 placental structure. on HE-stained paraffin sections, of the placental layers in female and males (F or M) from mother fed a control (CD) or high-fat (HFD) diet. (A) Details of the labyrinth layer. (B) Details of the junctional zone. Scale bars indicate 200 µm. (C) Measurement of the area and shape of the labyrinth and total placenta on histological slides. “Minor” and “major” indicate the width and length. Data are presented in arbitrary units, as means ± SEM (n = 8 per group). Non-parametric Kruskal-Wallis statistical tests indicated an absence of difference between the four groups for all measurements. (PDF) [file pone.0047986.s001.pdf]

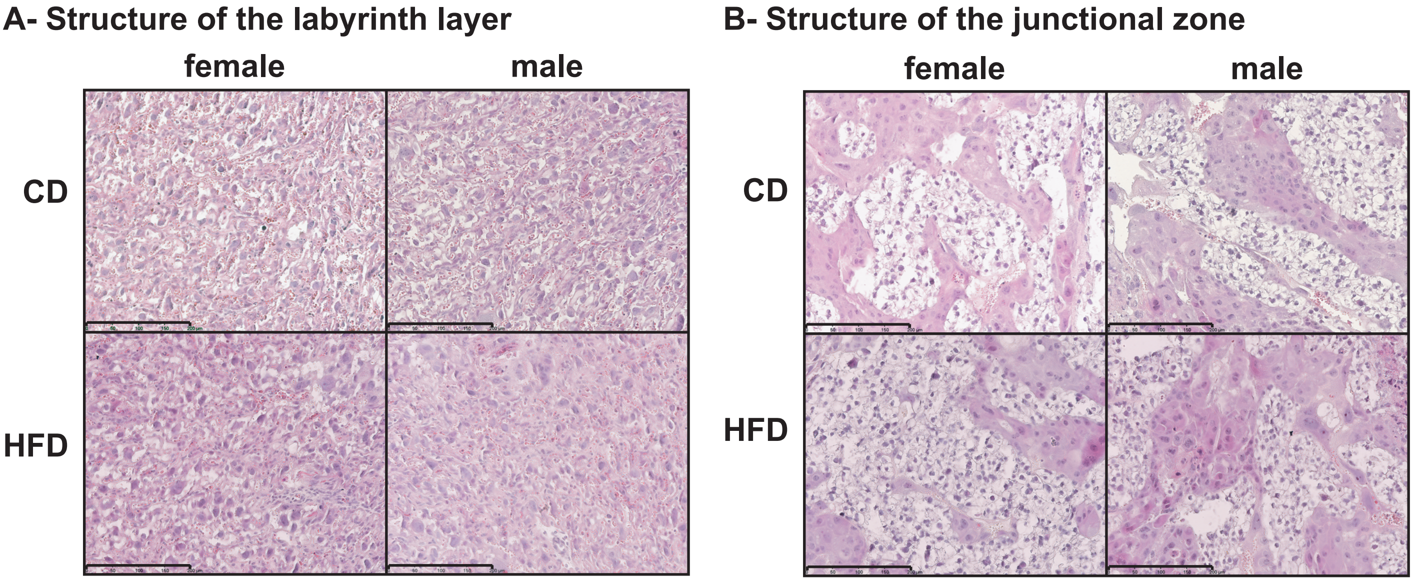

C-

	Labyrinth	Total	Labyrinth/total
**Surface**			
F CD	5.02 ± 0.37	10.90 ± 0.69	46% ± 0.8%
F HFD	4.74 ± 0.39	10.32 ± 0.93	46% ± 1.0%
M CD	4.87 ± 0.45	11.01 ± 0.96	44% ± 1.0%
M HFD	4.80 ± 0.28	10.63 ± 0.72	45% ± 0.9%
**Shape**			
Minor			
F CD	1193 ± 63	2147 ± 94	56% ± 1.3%
F HFD	1072 ± 58	2003 ± 109	54% ± 1.3%
M CD	1082 ± 57	2145 ± 104	50% ± 1.2%
M HFD	1110 ± 39	2138 ± 62	52% ± 0.9%
Major			
F CD	5391 ± 345	6470 ± 235	83% ± 2.5%
F HFD	5643 ± 360	6532 ± 364	86% ± 1.5%
M CD	5730 ± 383	6506 ± 349	88% ± 1.6%
M HFD	5645 ± 218	6486 ± 211	87% ± 1.5%

Supplementary figure S1: Analysis of E15.5 placental structure, on HE-stained paraffin sections, of the placental layers in female and males (F or M) from mother fed a control (CD) or high-fat (HFD) diet.

(A) Details of the labyrinth layer. **(B)** Details of the junctional zone. Scale bars indicate 200 µm. **(C)** Measurement of the area and shape of the labyrinth and total placenta on histological slides. “Minor” and “major” indicate the width and length. Data are presented in arbitrary units, as means ± SEM (n=8 per group). Non parametric Kruskal-Wallis statistical tests indicated an absence of difference between the four groups for all measurements.
