# Supplementary material for: Maternal Diets Trigger Sex-Specific Divergent Trajectories of Gene Expression and Epigenetic Systems in Mouse Placenta
Source: PLoS One. 2012 Nov 5;7(11):e47986. doi: 10.1371/journal.pone.0047986 (PMC3489896; doi:10.1371/journal.pone.0047986)
Supplement: Figure S2 — Histograms of epigenetic machinery expression levels in microarrays and RT-qPCR. Line indicates statistical significance and the number refers to log2 fold change. (PDF) [file pone.0047986.s002.pdf]

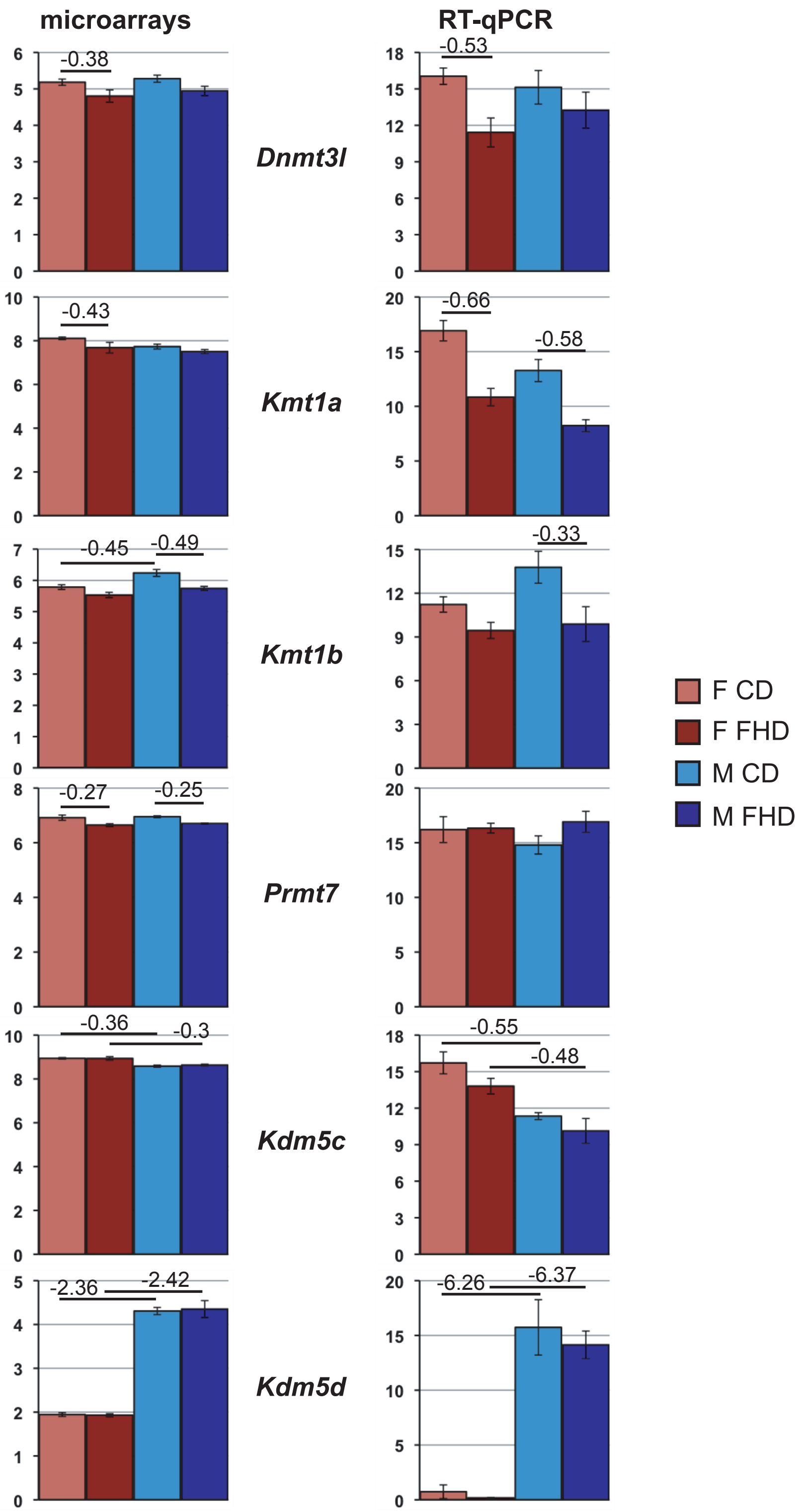

Supplemental figure S2: Histograms of epigenetic machinery expression levels in microarrays and RT-qPCR.
Line indicates statistical significance and the number refers to log2 fold change.
